# Activation of Persulfate for Improved Naproxen Degradation Using FeCo_2_O_4_@g-C_3_N_4_ Heterojunction Photocatalysts

**DOI:** 10.1021/acsomega.1c04896

**Published:** 2021-12-13

**Authors:** Baskaran Palanivel, Md Shahadat Hossain, Romulo R. Macadangdang, Chinnadurai Ayappan, Vignesh Krishnan, Raj Marnadu, Thirunavukarasu Kalaivani, Fahad A Alharthi, Gedi Sreedevi

**Affiliations:** † Department of Physics, Kings Engineering College, Sriperumbudur, Kancheepuram, Tamil Nadu 602117, India; ‡ Department of Innovation Systems Engineering, Graduate School of Engineering, Utsunomiya University, Yoto 7-1-2, Utsunomiya 321-8585, Japan; § Department of Medical Technology, Institute of Arts and Sciences, Far Eastern University, Manila 1008, Philippines; ∥ Department of Physics and Nanotechnology, SRM Institute of Science and Technology, Kattankulathur, Chengalpattu, Tamil Nadu 603203, India; ⊥ PG Department of Physics, GTN Arts College, Dindigul, Tamil Nadu 624005, India; # Chemistry Department, College of Science, King Saud University, Riyadh 1145, Saudi Arabia; ∇ School of Chemical Engineering, Yeungnam University, Gyeongsan 38541, Republic of Korea

## Abstract

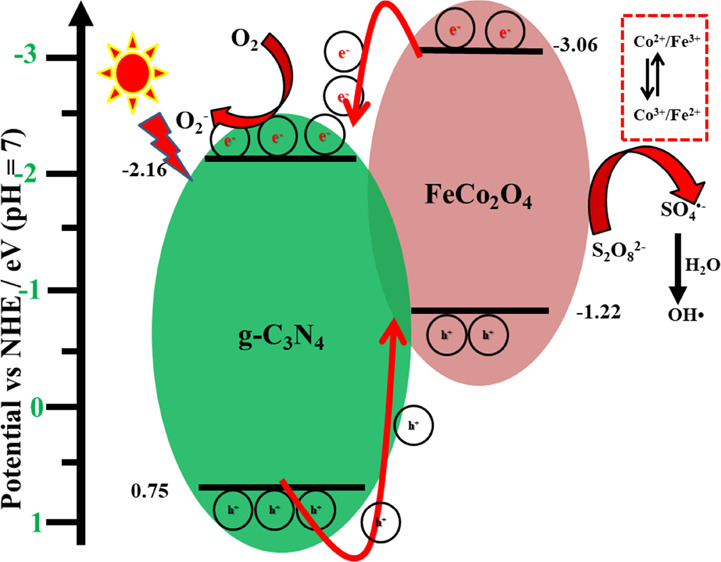

An effective heterojunction with robust charge separation and enormous degradation efficiency is the major task for photocatalyst preparation. In this study, we have prepared the FeCo_2_O_4_-loaded g-C_3_N_4_ nanosheet by the sol–gel-assisted calcination method for photo-Fenton-like degradation under visible-light irradiation by activating persulfate. The nanocomposite exhibits a higher charge separation efficiency than pure g-C_3_N_4_ and FeCo_2_O_4_ for the degradation reaction against naproxen drugs. An effective interaction between the nanoparticles increases the degradation efficiency up to 91% with a synergistic index of 73.62%. Moreover, the nanocomposite exhibits a 78% mineralization efficiency against the naproxen pollutant under visible-light irradiation. For practical implementation, the degradation reaction was tested with various pH values, different water sources (DI, lake, and tap water), and light sources (LED (visible)/direct sunlight (UV–visible)). Moreover, the possible degradation mechanism predicted by the elemental trapping experiment and the recycling experiment clearly revealed that the heterojunction composite has a high enough degradation stability.

## 1 Introduction

The continuous discharge of a range of organic effluents from different industries imposes environmental threat and affects human health. Water is considered a prime need for humans, but the quality of water is not adequate for the basic need of human lives. The level of wastewater has increased with industrialization, such as paper mills, textiles, plastic, concrete, and medicines. Especially, dyeing and paper industries produce wastewater, which contains toxic aromatic dyes and it is difficult to degrade them due to their stable chemical structure.^1^ A variety of methods, for example, biological treatment, electrochemical treatment, adsorption, and advanced oxidation process (AOP) have been developed to degrade organic waste. Among these purification methods, the AOP is an effective method to eliminate the organic wastes from industrial outlets. In particular, the persulfate (PS)-activated AOP for treating the industrial pollutants has been paid much attention over the recent years due to its production of highly active sulfate (SO_4_•^–^) radicals. These radicals have several advantages, which include a higher redox potential (2.5–3.1 V) than hydroxyl radicals (1.9–2.7 V), a broad range of selectivity, a longer lifetime, and applicability for a wide range of pH values. PS can be activated by several ways, including the heat process, UV radiation, ultrasound treatment, and by transition metal ions (Fe, Co, Mn, Cu, etc.). In particular, cobalt ions can be used to as an effective material for PS activation due to their higher standard redox potential (1.83 V) than any other metal ions (Fe = 0.77 V, Cu = 0.16 V). Therefore, the research focuses on cobalt-based metal oxides for PS activation toward organic pollutant degradation.^2,3^

Recently, cobalt based spinel metal oxides have been extensively used in catalytic reactions due to their unique electronic, optical, physical, and chemical properties. Especially, FeCo_2_O_4_ has received great attention due to its exceptional electrochemical behavior, great structural constancy, eco-friendliness, low metal ion discharge, and Fe/Co interaction. However, particle agglomeration due to the high surface energy of FeCo_2_O_4_ intensely reduces its reactive sites and specific surface area.^4,5^ To overcome this drawback, a heterojunction has been formed with FeCo_2_O_4_ using carbon-based materials to enhance the photocatalytic activity.^6^ In general, the heterojunction formation between the nanoparticles helps to retain the redox potential of each catalyst and also helps to induce charge separation.^7^ Moreover, the heterojunction can extend the light absorption range to some extent.^8,9^ In particular, g-C_3_N_4_ has been considered as an effective 2D visible-light photocatalyst due to its stable structure, facile preparation, unique electronic structure, and high specific surface area. In addition, polymeric g-C_3_N_4_, considered as an exciting sustainable material due to its unique physiochemical properties, acts as a catalyst for organic pollutant degradation with an appropriate band gap value of 2.7 eV.^10^ Moreover, the long range of the π–π conjugation system presented in g-C_3_N_4_ gives it a stable allotrope form under ambient environmental conditions.^11^ The combination of FeCo_2_O_4_/g-C_3_N_4_ enhances the photocatalytic activity of dye degradation compared to their pure material forms as reported by Zhao et al.^2^

Based on the literature reports, we have synthesized the FeCo_2_O_4_/g-C_3_N_4_ heterojunction by a single step sol–gel assisted calcination method and it was applied to photo-Fenton-like naproxen degradation by activating PS under visible-light irradiation. Furthermore, the photocatalytic activity under different environments was also performed and the reaction mechanism and the stability test were also conducted.

## 2 Results and Discussion

### 2.1 Characterization of Catalysts

**Figure 1:**
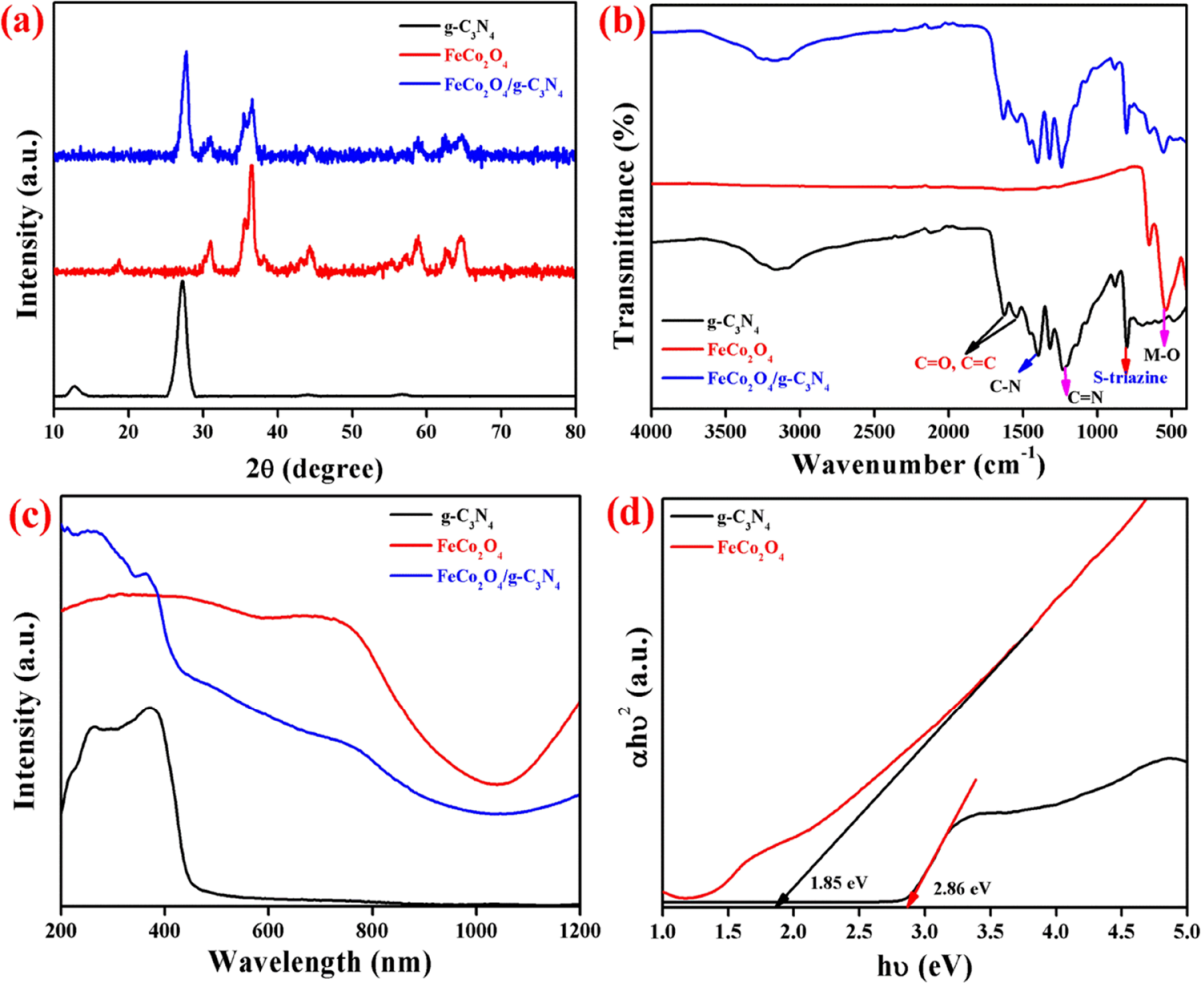
(a) XRD patterns, (b) FTIR spectra, (c) UV–vis spectra, and (d) Tauc’s plot.

The crystal phase of the prepared photocatalysts was analyzed by X-ray diffraction (XRD) patterns, and the results are shown in Figure 1a. The two peaks of g-C_3_N_4_ positioned at 12.64° and 27.22° showed that the planes (100) and (002) were related to the distance of the interlayer structural package and separation of conjugated aromatic systems, respectively.^12^ The characteristic peaks of spinel FeCo_2_O_4_ positioned at 18.75°, 30.96°, 36.55°, 44.29°, 58.87°, and 64.59° corresponding to the (111), (220), (311), (400), (511), and (410) planes, respectively, were well-matched with previous reports, and it is well-suited with the standard JCPDS card 80–1534 with a *Fd*3*m* space group.^4,6^ From the XRD pattern, it is clear that the composite consists of g-C_3_N_4_ and FeCo_2_O_4_ diffraction peaks. A close observation of composite XRD peaks reveals the slight peak shift in the (002) plane of g-C_3_N_4_ toward the higher angle side and no diffraction peak shift in FeCo_2_O_4_. This result indicated that the FeCo_2_O_4_ nanoparticles were deposited on g-C_3_N_4_ in the nanocomposite and it reduces the d-space of conjugated aromatic systems.^13^

The vibrational behavior of the photocatalysts was analyzed by Fourier transform infrared (FTIR) spectroscopy and the recorded spectra are shown in Figure 1b. g-C_3_N_4_ shows characteristic peaks of the triazine unit, C–N heterocycles, and surface-adsorbed water molecules in the frequency ranges of 802 cm^–1^, 1220–1650 cm^–1^, and 3000–3300 cm^–1^, respectively.^13^ The transmittance peaks at 540 cm^–1^ and 650 cm^–1^ in FeCo_2_O_4_ nanoparticles reveal the presence of metal–oxygen (M–O) stretching vibration. The composite photocatalyst has the FTIR spectrum of both g-C_3_N_4_ and FeCo_2_O_4_, which represents the formation of a heterojunction between the nanoparticles. Moreover, the peak shift of the triazine unit and the M–O bond in the nanocomposite confirms the chemical interaction between the nanoparticles.^2^

Light absorption ability of the prepared photocatalysts was studied by UV–vis absorption spectra, which are shown in Figure 1c. From the absorption spectra, one can conclude that the prepared nanoparticles exhibit the visible-light absorption behavior, and the absorption spectrum was observed in the 400–800 nm region. Pure g-C_3_N_4_ has an absorption wavelength of 450 nm and FeCo_2_O_4_ exhibits the absorption in the range of 730 nm, which describes the π–π*/*n*–π* and O_2_^–^–Co_3_^+^ electron transactions, respectively.^2,14^ The nanocomposite FeCo_2_O_4_/g-C_3_N_4_ shows an enhanced visible-light absorption ability in comparison with g-C_3_N_4_ and exhibits higher absorption intensity compared to pure nanoparticles. These results described the strong interaction between the nanoparticles, and it was well-matched with the XRD and FTIR results.^14^ The band gap of g-C_3_N_4_ and FeCO_2_O_4_ has been calculated by Tauc’s plot, and the result is shown in Figure 1d. The calculated band gap (*E*_g_) of g-C_3_N_4_ and FeCo_2_O_4_ was 2.86 and 1.85 eV, respectively.

**Figure 2:**
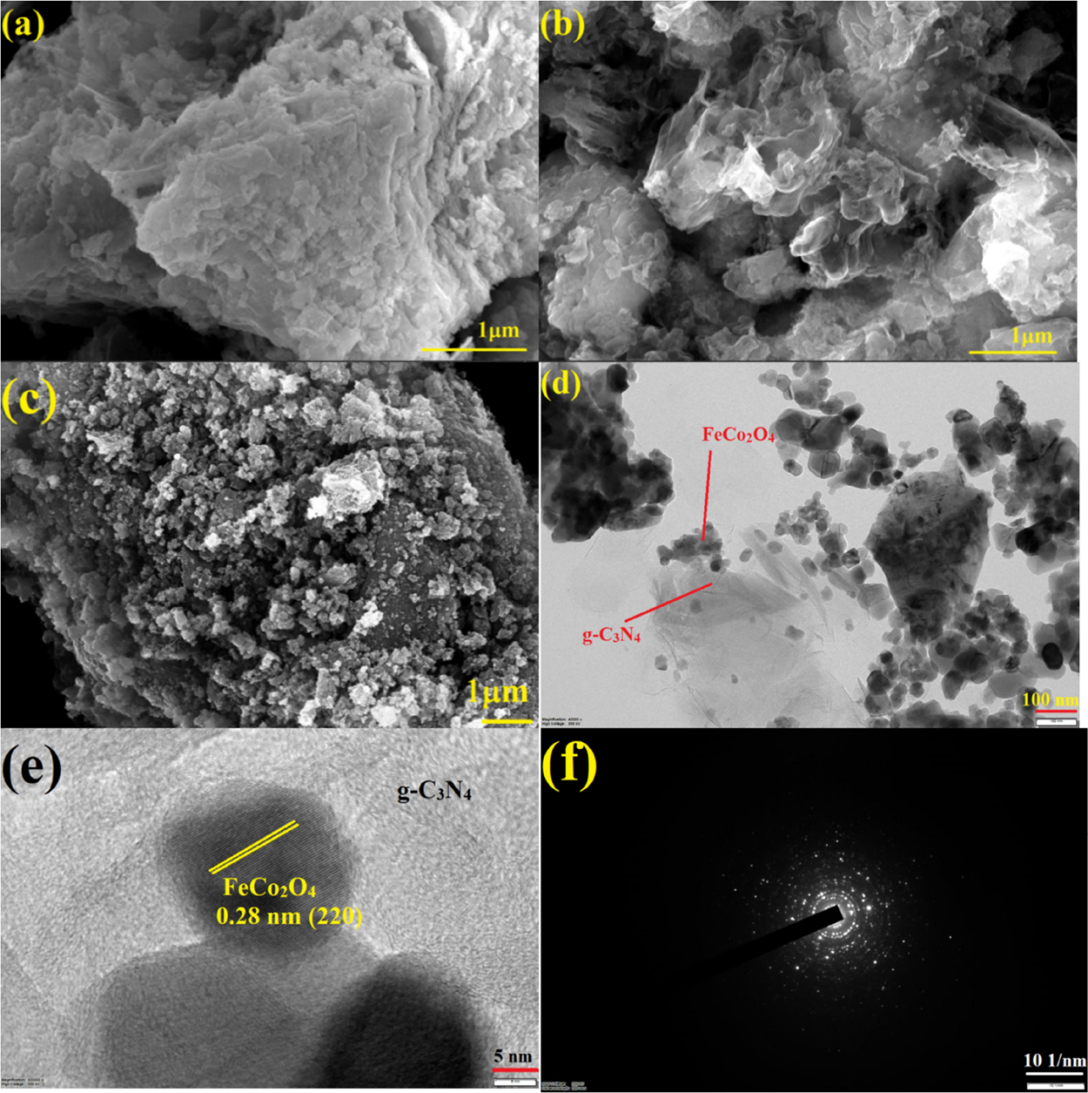
FESEM images of (a) FeCo_2_O_4_, (b) g-C_3_N_4_, and (c) FeCo_2_O_4_/g-C_3_N_4_. TEM image of FeCo_2_O_4_/g-C_3_N_4_ (d) and (e) HRTEM and (f) SAED patterns.

**Figure 3:**
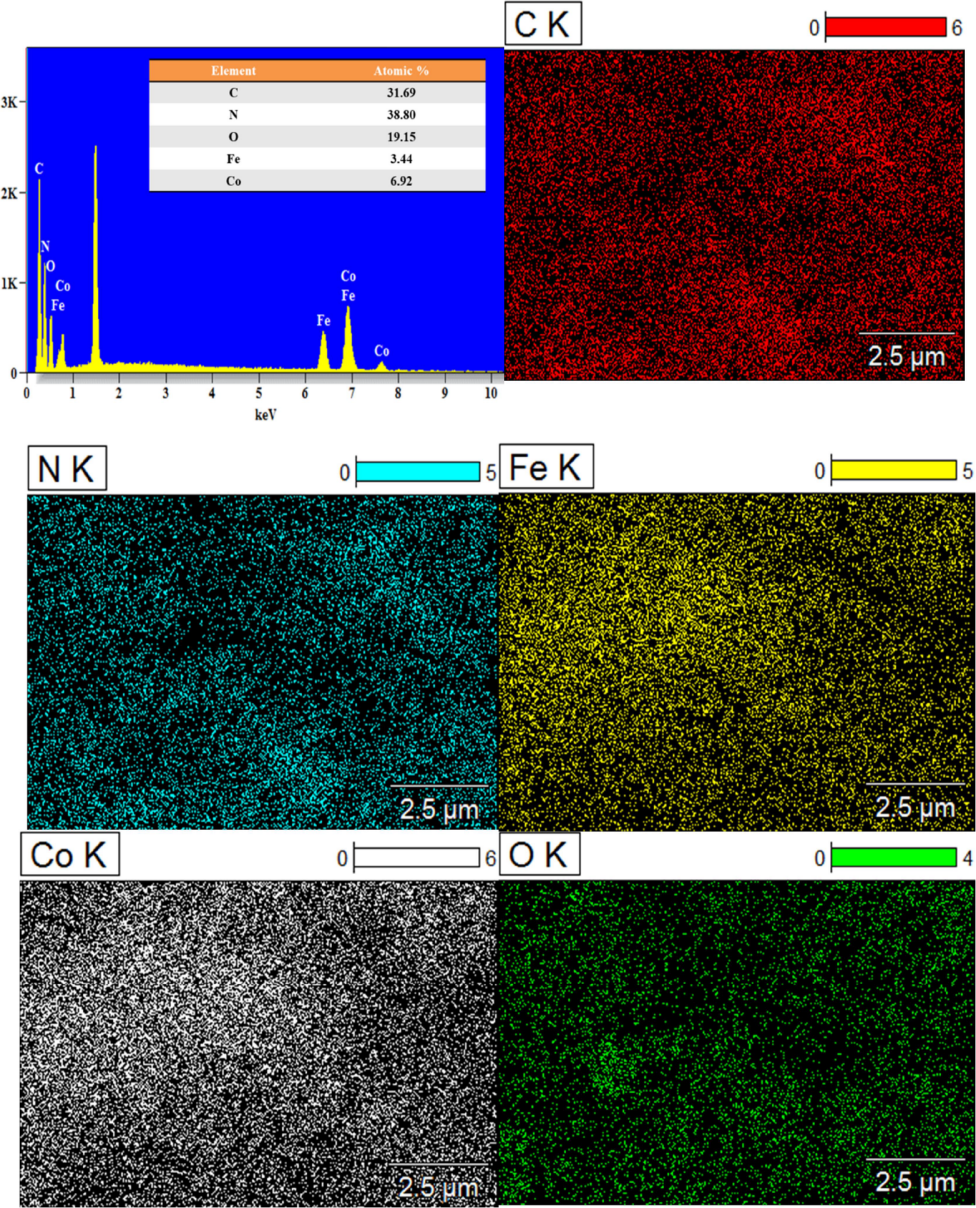
EDS spectrum and elemental mapping of FeCo_2_O_4_/g-C_3_N_4_.

Figure 2 reveals the morphological nature of the prepared photocatalysts. As shown in the field-emission scanning electron microscopy (FESEM) micrographs, it can be noticed that FeCo_2_O_4_ displays an agglomerated particle-like structure and g-C_3_N_4_ exhibits sheetlike morphology. The FESEM image of the nanocomposite confirmed that the g-C_3_N_4_ nanosheet was completely covered by FeCo_2_O_4_ nanoparticles and it makes the heterojunction formation. Furthermore, the nanocomposite formation was confirmed by transmission electron microscopy (TEM) analysis, and the results are displayed in Figure 2d–f. The deposition of FeCo_2_O_4_ nanoparticles on the g-C_3_N_4_ nanosheet was clearly observed from TEM analysis, whereas the high-resolution TEM (HRTEM) image clearly shows the heterojunction between the nanoparticles and it shows a d-space value of 0.28 nm related to the FeCo_2_O_4_ (220) plane. In addition, the elements presented in the nanocomposite and their distribution was investigated by energy-dispersive spectrometry (EDS) spectra and elemental mapping analysis. The presence of carbon (C), nitrogen (N), oxygen (O), iron (Fe), and cobalt (Co) atoms in the FeCo_2_O_4_/g-C_3_N_4_ nanocomposite with an atomic percentage of 31.69, 38.80, 19.15, 3.44, and 6.92%, respectively, is confirmed in Figure 3. Hence, one can conclude that the heterojunction formation exists between the g-C_3_N_4_ and FeCo_2_O_4_ nanoparticles.

**Figure 4:**
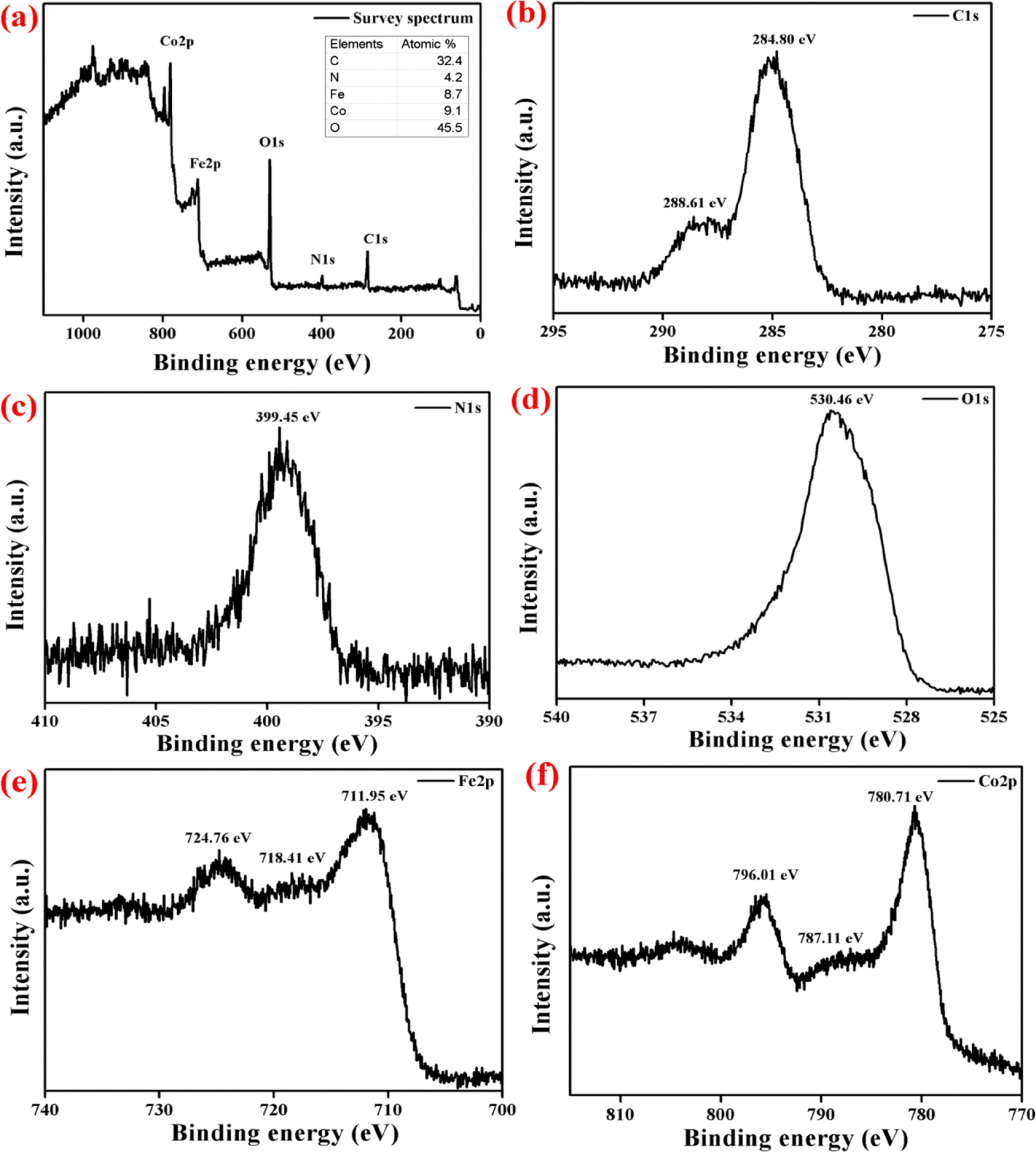
XPS analysis of FeCo_2_O_4_/g-C_3_N_4_. (a) Survey spectrum, (b) C 1s, (c) N 1s, (d) O 1s, (e) Fe 2p, and (f) Co 2p.

Further the chemical interaction between the nanoparticles was investigated by X-ray photoelectron spectroscopy (XPS) analysis, and the results are shown in Figure 4. From the survey spectrum, the presence of all elements in the nanocomposite can be confirmed, and this was well-matched with EDS and elemental mapping analysis. The high-resolution XPS (HRXPS) spectrum of each element is shown in Figure 4b–f. The HRXPS spectrum of C 1s consists of the peaks related to C–C interaction and C–N interaction positioned at 284.8 and 288.61 eV, respectively.^14^ The HRXPS peak positioned at 399.45 in the N 1s spectrum revealed the triazine unit in g-C_3_N_4_ with sp^2^-hybridized C–N interaction. The O 1s HRXPS spectrum consists of a major peak positioned at 530.46 eV indicating the lattice oxygen in spinel FeCo_2_O_4_.^14^Figure 4e shows the HRXPS spectrum of Fe 2p and it has three peaks positioned at 711.95, 718.41, and 724.76 eV attributed to Fe2p_3/2_, the satellite peak, and Fe2p_1/2_, respectively, and these indicate the presence of Fe^3+^ ions in the nanocomposite. The Co 2p spectrum has two major peaks (780.71 and 796.01 eV) associated with the satellite peak (787.11 eV), revealing the presence of Co^2+^ ions in the composite photocatalyst.^3^

### 2.2 Photocatalytic Activities

**Figure 5:**
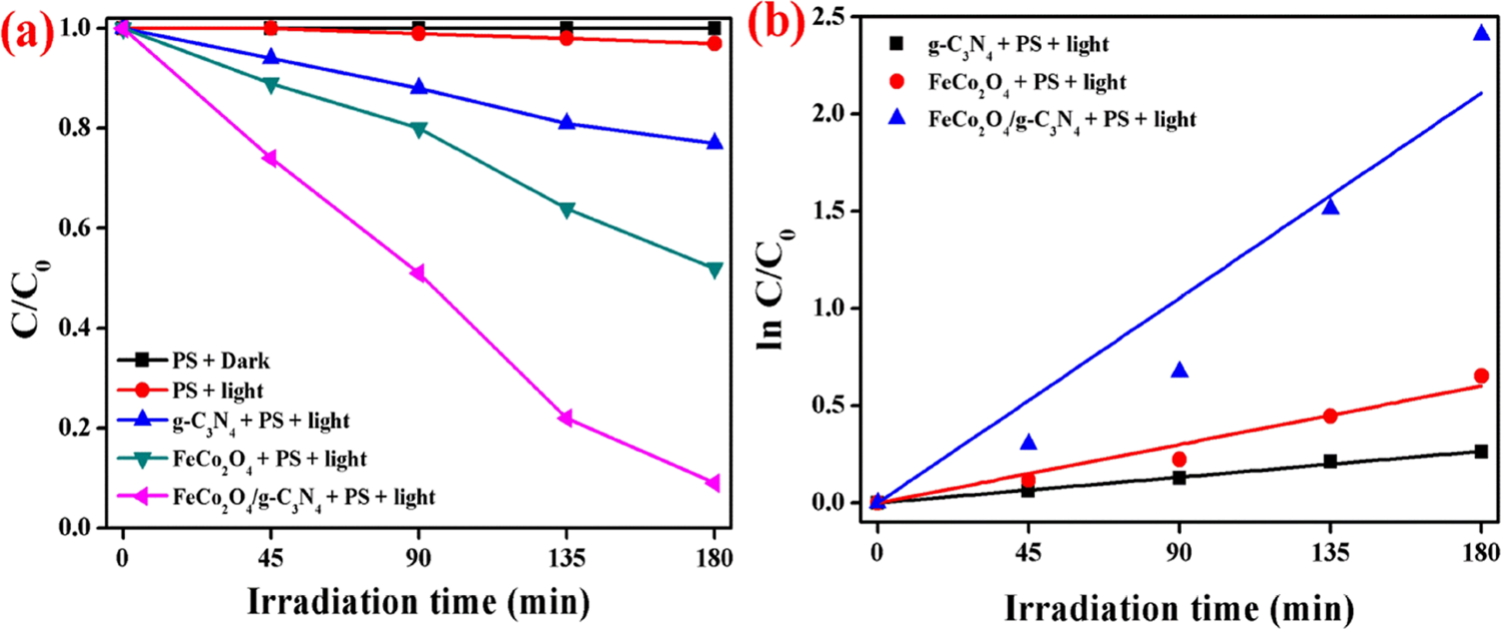
Naproxen degradation. (a) Degradation plot and (b) pseudo-first-order kinetic plot (reaction conditions: catalysts, 50 mg; naproxen concentration, 5 mg/L; PS, 0.25 g/L; pH, 4.2).

The photocatalytic ability of the prepared nanoparticles was investigated through naproxen degradation by activating PS under visible-light irradiation, and the results of degradation and their kinetic plot are shown in Figure 5a,b. Naproxen degradation was negligible under the dark and light conditions without addition of photocatalysts, and this result reveals the stability of the pollutant. Moreover, the photolysis process exposed that the activation of PS and degradation takes place with the addition of photocatalysts. The maximum naproxen degradation achieved was up to 91% by activating PS using the FeCo_2_O_4_/g-C_3_N_4_ composite photocatalyst. The PS-activated naproxen degradation efficiency of g-C_3_N_4_ and FeCo_2_O_4_ was 23% and 48% under visible-light irradiation. To determine the synergistic index (SI) of the photocatalyst with PS for naproxen degradation, the photocatalytic experiment was conducted without the addition of PS under light conditions, and the degradation result is shown in Figure S1. The naproxen degradation efficiency recorded for g-C_3_N_4_, FeCo_2_O_4_, and FeCo_2_O_4_/g-C_3_N_4_ was 13, 11, and 21%, respectively. The SI has been calculated using the following relation^3^
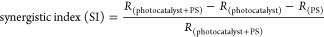
where *R* represents the pollutant degradation efficiency. The calculated SI values of each prepared photocatalysts were tabulated. From Table S1, it can be confirmed that the composite FeCo_2_O_4_/g-C_3_N_4_ has a higher SI value of 73.62% against naproxen degradation in comparison with g-C_3_N_4_ (30.43%) and FeCo_2_O_4_ (72.92%). Hence, this result clearly revealed that the synergistic interaction between the nanocomposite and PS helps to improve naproxen degradation under visible-light irradiation. Furthermore, the rate (*k*) of naproxen degradation was estimated by a pseudo-first-order kinetic plot, and it is shown in Figure 5b. The estimated “*k*” value of g-C_3_N_4_, FeCo_2_O_4_, and FeCo_2_O_4_/g-C_3_N_4_ was 0.001, 0.003, and 0.013 min^–1^, respectively.

**Figure 6:**
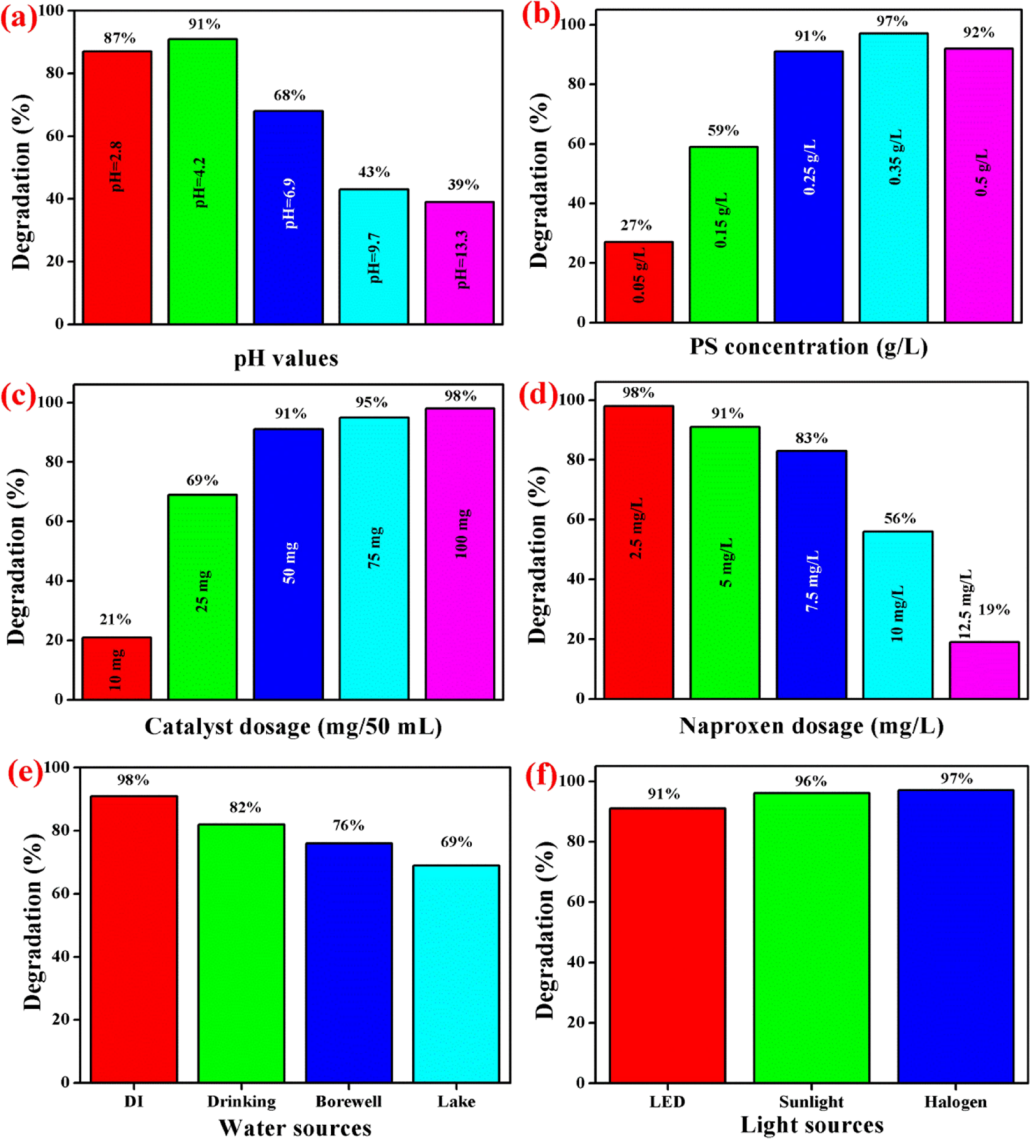
Naproxen degradation under different environmental conditions. (a) Initial pH of the solution, (b) PS concentration, (c) photocatalyst dosage, (d) naproxen concentration, (e) water sources for the degradation reaction, and (f) different light source irradiation (unless stated otherwise, the reaction conditions are: catalysts, 50 mg; naproxen concentration, 5 mg/L; PS, 0.25 g/L; pH, 4.2; water source: DI water).

The naproxen degradation efficiency under different environmental conditions were studied and are portrayed in Figure 6. The initial pH value of the solution plays a major role for naproxen degradation. Therefore, a series of degradation experiments were conducted using FeCo_2_O_4_/g-C_3_N_4_ nanocomposites under various pH values ranging from ∼3 to 13 and the results are shown in Figure 6a. Maximum degradation efficiency was achieved at a pH value of 4.2, which is gradually reduced while increasing the initial pH value of the solution, and it shows a minimum degradation efficiency of 39% at pH = 13.3. At higher pH conditions, the •OH radicals, OH^–^, and SO_5_^2–^ may accumulate on the surface of the catalysts, which reduces the conversion of •SO_4_^–^ from •SO_5_^–2^.^3^ On the other hand, very high acidic conditions also decrease the naproxen degradation efficiency due to the trapping effect on sulfate and hydroxyl radicals by protons.^5^ From Figure 6b, the naproxen degradation under various PS concentrations can be observed. It was clearly noticed that the degradation efficiency increases with the increase in the PS concentration from 0.05 to 0.35 g/L and the efficiency decreased further on increasing the concentration of PS to about 0.5 g/L. This result indicated that the higher PS concentration may lead to self-quenching reactions, which affect pollutant degradation.^3^Figure 6c shows the naproxen degradation under various concentrations of the photocatalyst. From the graph, it was clearly noticed that the naproxen degradation efficiency was improved with the increment of the catalyst dosage and it reached 98% degradation efficiency using 100 mg of photocatalysts. An efficiency increment reveals the availability of more reactive sites for naproxen degradation reactions upon the catalyst dosage increment.^5^ The effect of naproxen concentration against the degradation efficiency test was conducted, and the result is displayed in Figure 6d. From the graph, it can be concluded that the degradation efficiency was reduced while increasing the pollutant concentration and it demonstrated the insufficient radical formation and inhibition of light utilization due to higher pollutant concentration.^14^

For real-world application, it is necessary to find the photocatalytic efficiency of the catalysts under various water environments. Therefore, the naproxen photodegradation experiment was conducted using drinking water, borewell water, and lake water, and the results are displayed in Figure 6e. From the results, it can be noted that the degradation efficiency using various water sources was quite low compared to deionized (DI) water. The presence of various anions and cations in the real-world water sources may affect the active sites of the photocatalyst, which can reduce the naproxen degradation efficiency.^15^ In addition, the various light sources also involved in naproxen degradation apply the photocatalyst for practical application. As shown in Figure 6f, one can conclude that the naproxen degradation efficiency was improved under the sunlight/halogen light irradiation in comparison to LED. This result may attribute to comparatively higher intensity than LED light.^16^

**Figure 7:**
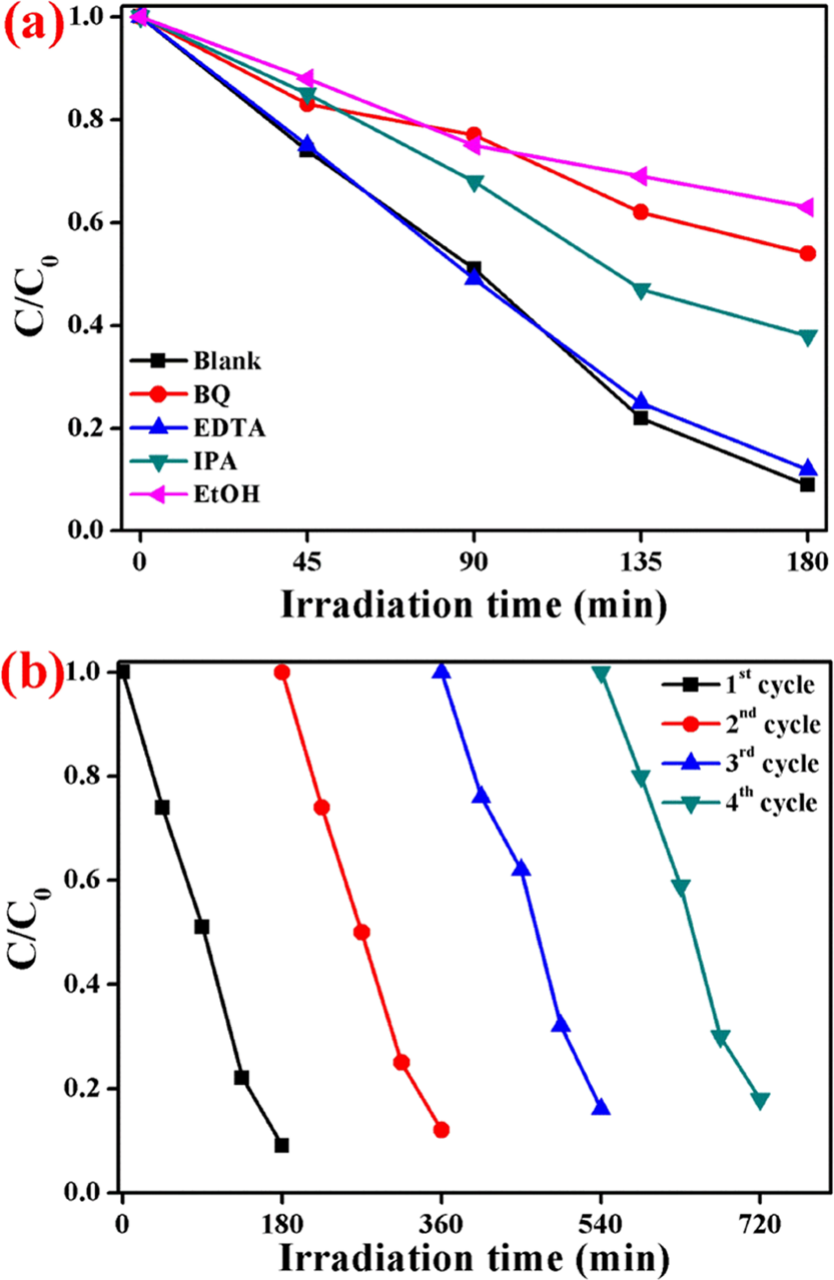
(a) Scavenger experiment and (b) recycle test (reaction conditions: catalysts, 50 mg; naproxen concentration, 5 mg/L; PS, 0.25 g/L; pH, 4.2).

To find the reactive radicals, which are involved in naproxen degradation, the scavenger experiment was conducted, and the result is shown in Figure 7a. From the trapping experiment, it was clearly noted that the addition of ethanol (EtOH) and benzoquinone (BQ) reduces the naproxen degradation to 37 and 46%, respectively. This result reveals that the SO_4_•^–^ and O_2_•^–^ radicals are the major contributor for the degradation reaction. In addition, IPA also suppresses the degradation efficiency to 68%, and it reveals that the hydroxyl radicals are a minor contributor for the photocatalytic degradation reaction. The addition of ethylenediamine tetraaceticacid (EDTA) does not alter the naproxen degradation efficiency and it results in the noncontribution of holes for the degradation reaction. Furthermore, the stable nature of the FeCo_2_O_4_/g-C_3_N_4_ photocatalysts was analyzed through the recycling experiment, and the result is shown in Figure 7b. Even after the fourth recycle, the photocatalyst showed a degradation efficiency of 82% and exhibited structural stability, which was confirmed by XRD (Figure S2) analysis. In addition to this, the mineralization efficiency of the FeCo_2_O_4_/g-C_3_N_4_ photocatalyst was also analyzed by a TOC (total organic carbon) analyzer, and the result is displayed in Figure S3. The nanocomposite photocatalyst showed a 78% TOC removal efficiency against naproxen pollutants under visible-light irradiation. Effective photocatalytic degradation behavior of the nanocomposite mainly depends on the charge separation process, which was examined by photoluminescence analysis, and the result is shown in Figure S4. The FeCo_2_O_4_/g-C_3_N_4_ nanocomposite exhibits lower PL emission intensity compared to pure g-C_3_N_4_ at room temperature analysis, and this result clearly demonstrated the inhibition of the charge recombination rate of the nanocomposite.^17^ Furthermore, the electron transport behavior and charge resistance of the prepared photocatalysts were analyzed by transient photocurrent measurements and electrochemical impedance (EIS) spectra, which are shown in Figure S5.^18,19^ From the photocurrent and EIS analysis, it can be concluded that the FeCo_2_O_4_/g-C_3_N_4_ photocatalyst exhibits a higher photocurrent response with a lower charge resistance than pure g-C_3_N_4_ and FeCo_2_O_4_ nanoparticles. This result clearly revealed that the heterojunction between the nanoparticles enhances the light harvesting behavior and charge carrier production.^18^ On the other hand, the smaller EIS arc size of the nanocomposite compared to pure nanoparticles reflects the lower charge transfer resistance, which helps to improve the electron–hole separation and transportation to promote the photocatalytic reaction.

**Figure 8:**
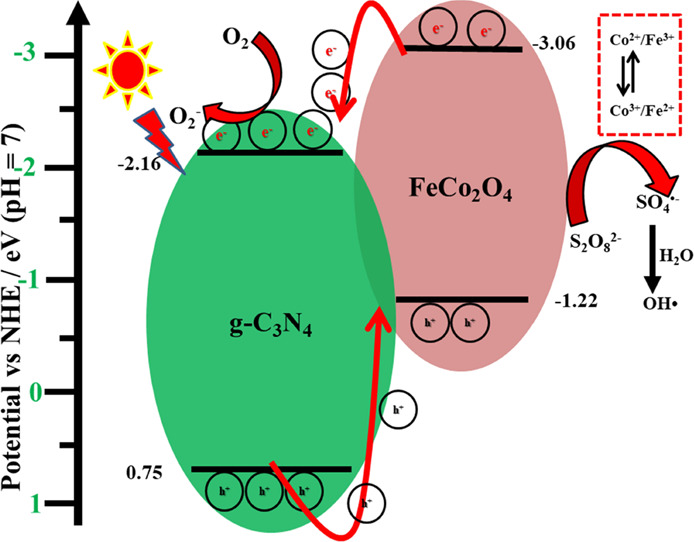
Possible reaction mechanism for naproxen degradation.

To propose the possible photodegradation mechanism, it is necessary to evaluate the band potential of the photocatalysts. Therefore, the valence band XPS (VBXPS) analysis was performed, and the obtained spectrum is shown in Figure S6. From the VBXPS results and *E*_g_ values, the conduction band potential of the prepared g-C_3_N_4_ and FeCo_2_O_4_ photocatalysts was calculated. The CB/VB values of g-C_3_N_4_ and FeCo_2_O_4_ were −2.16/0.75 and −3.06/–1.22 V, respectively. The possible reaction charge transfer pathway and the radical production mechanism for naproxen photodegradation are illustrated in Figure 8. Upon visible-light irradiation, both the nanoparticles generated charge pairs. Due to the potential differences between the nanoparticles, the electrons and holes are migrated and accumulated separately on the CB of g-C_3_N_4_ and the VB of FeCo_2_O_4_. Then, electrons on the CB of g-C_3_N_4_ generate the superoxide radicals, while holes in the VB of FeCo_2_O_4_ do not contribute in the degradation reaction, which was confirmed by the scavenger test. On the other hand, the photogenerated electrons in the CB of FeCo_2_O_4_ also activate the PS to generate the highly active SO_4_•^–^ and OH• radicals by the photoreduction process. In this way, the photo-Fenton-like reaction was maintained to degrade the naproxen pollutant under visible-light irradiation. The photo-Fenton-like naproxen degradation reaction was proposed as follows:^2,14,17^
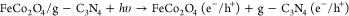

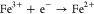

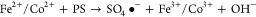

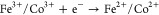

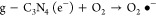

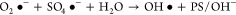

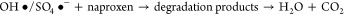


## 3 Conclusions

An effective FeCo_2_O_4_/g-C_3_N_4_ heterojunction was successfully synthesized by following a sol–gel calcination process, and it was applied for naproxen degradation by PS activation. The heterojunction between the nanoparticles helped improve the light absorption ability and enhance the charge separation efficiency compared to pure nanoparticles. This synergistic interaction between the nanoparticles produced 91% naproxen degradation under visible-light irradiation by PS activation. Moreover, the photocatalyst shows higher stability and is suitable for practical usage under various environmental conditions. Hence, this nanocomposite FeCo_2_O_4_/g-C_3_N_4_ could be an efficient candidate for environmental remediation application.

## 4 Experimental Section

### 4.1 Synthesis Procedure of Photocatalysts

For FeCo_2_O_4_/g-C_3_N_4_ synthesis, a 1:2 mmol ratio of iron nitrate nonahydrate and cobalt nitrate hexahydrate was dissolved in 10 mL of ethanol. Then, 3 mmol citric acid was added to the solution. The solution was magnetically stirred until the gel was formed. Then, 5 g of melamine was added to the gel and stirred for another 30 min. Then, the gel was dried in a hot air oven at 120 °C for 2 h. The dried sample was calcined to 550 °C for 2 h in a muffle furnace. The same procedure was followed to prepare FeCo_2_O_4_ without the addition of melamine. Pure g-C_3_N_4_ was prepared by direct calcination of melamine at 550 °C for 2 h.

## Supporting Information Available

Characterization methods; photocatalytic activity test; naproxen degradation plot without PS; XRD analysis for pure and recycled samples; TOC analysis; PL analysis; photocurrent and impedance analysis; and VBXPS spectrum (PDF)
