# Supplementary material for: Activation of Persulfate for Improved Naproxen Degradation Using FeCo2O4@g-C3N4 Heterojunction Photocatalysts
Source: ACS Omega. 2021 Dec 13;6(50):34563–71. doi: 10.1021/acsomega.1c04896 (PMC8697371; doi:10.1021/acsomega.1c04896)
Supplement: Supplementary file 1 — ao1c04896_si_001.pdf [file ao1c04896_si_001.pdf]

Supporting Information

Activation of Persulfate for Improved Naproxen Degradation using $FeCo_2O_4$@g-C_3N_4 Heterojunction Photocatalyst

Baskaran Palanivel [1], Md Shahadat Hossain [2], Romulo R. Macadangdang Jr [3], Chinnadurai Ayappan [4], Vignesh Krishnan [4], Raj Marnadu [5], Thirunavukarasu Kalaivani [4], Fahad A Alharthi [6], Gedi Sreedevi [7]**

1. ***Department of Physics, Kings Engineering College, Sriperumbudur, Kancheepuram – 602117, Tamil Nadu, India***
2. ***Department of Innovation Systems Engineering, Graduate School of Engineering, Utsunomiya University, Yoto 7-1-2, Utsunomiya 321-8585, Japan***
3. ***Department of Medical Technology, Institute of Arts and Sciences, Far Eastern University, Manila, Philippines***
4. ***Department of Physics and Nanotechnology, SRM Institute of Science and Technology, Kattankulathur, Chengalpattu – 603203, Tamil Nadu, India***
5. ***PG Department of Physics, GTN Arts college, Dindigul - 624 005, Tamilnadu, India***
6. ***Chemistry Department, College of Science, King Saud University, Riyadh 1145, Saudi Arabia***
7. ***School of Chemical Engineering, Yeungnam University, Gyeongsan, 38541, Republic of Korea***

Corresponding authors

Baskaran Palanivel: baskaranpj1993@gmail.com

Gedi Sreedevi: drsrvi9@gmail.com

Characterization methods

The crystalline nature of the materials was analyzed by PXRD (powder X-ray diffractometer) using PANaltical X'pert pro (Cu Kα – 1.5405Å). The optical absorption properties of the nanoparticles were obtained by UV-DRS spectra using Shimadzu UV3600+ spectrometer with the range of 200 - 1200 nm. The FESEM (field emission scanning electron microscope) and TEM (transmission electron microscope)analyses of nanocatalysts were carried out by Thermosceintific Apreo S and JEOL, JEM 2100 plus with electron accelerating voltage of 20 kV and 200 kV respectively. PL (Photoluminescence) spectra of as-prepared nanoparticles were obtained by the HORIBA Fluorolog instrument with a laser excitation wavelength of 396 nm. Fourier transform infrared spectroscopy analysis of the prepared nanoparticles was performed by Shimadzu, IRTRACER 100 with ATR mode.

Photocatalytic activity test

The homemade Pyrex-glass reactor with water circulating cooling system was used for the photocatalytic degradation test. The photocatalytic activity of the prepared samples was examined by degradation of commercial naproxen drug under 40W LED lamp ($\lambda > 400$ nm (31,619 lux)). In typical, the synthesized photocatalysts (50 mg) was dispersed to the 100 ml of naproxen (5 mg/L) solutions. The photocatalytic process was carried out at ambient pressure and temperature (~ 25-27 °C). To attain the organic pollutant adsorption and desorption equilibrium at the photocatalyst surface, the solution was stirred for 30 min. Then the solution pH was adjusted to acetic/base condition using diluted HCl/NaOH solution. Followed by 25 mg of potassium persulfate was added into the solution and it is exposed under LED illumination to initiate the photocatalytic process. At regular intervals of time, 3 ml of pollutant solution was taken out and examine by UV-Vis spectroscopy (Shimadzu UV3600+). The elemental trapping experiment was conducted by the addition of scavengers to the MB solution under the light irradiation. The benzoquinone (BQ), isopropanol (IPA), ethanol (EtOH) and disodium-ethylenediaminetetraacaiticacid (EDTA-2A) were used as the scavengers for the O_2^-, OH• $SO_4^{\bullet -}$, and h^+ radicals respectively.

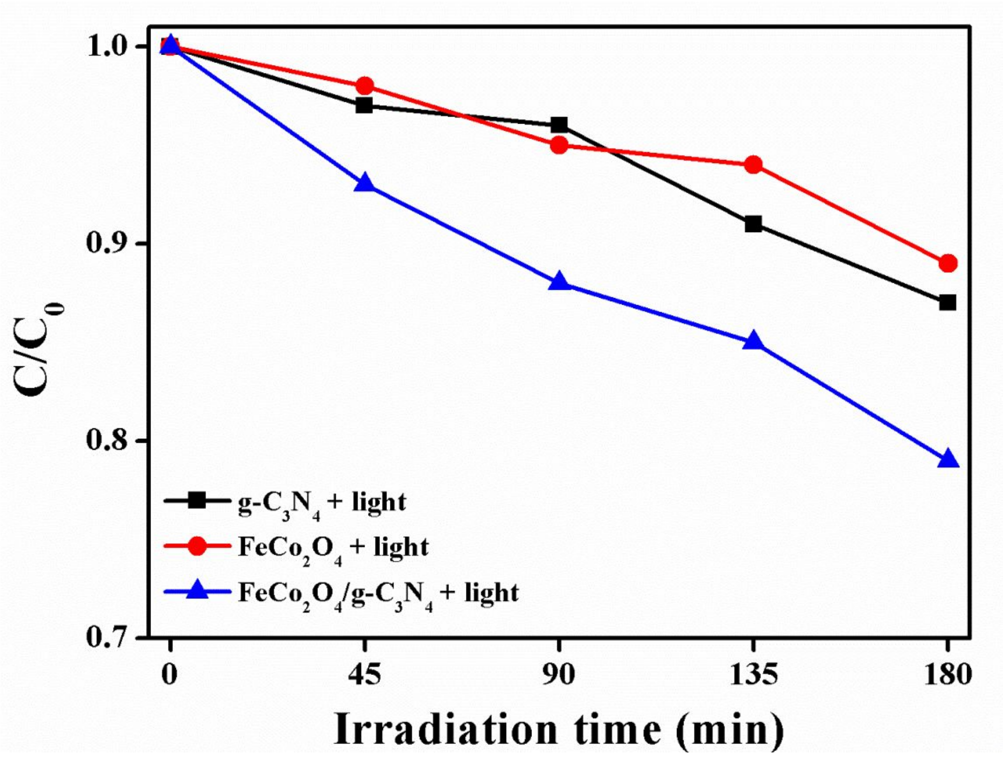

Figure S1. Naproxen degradation without PS

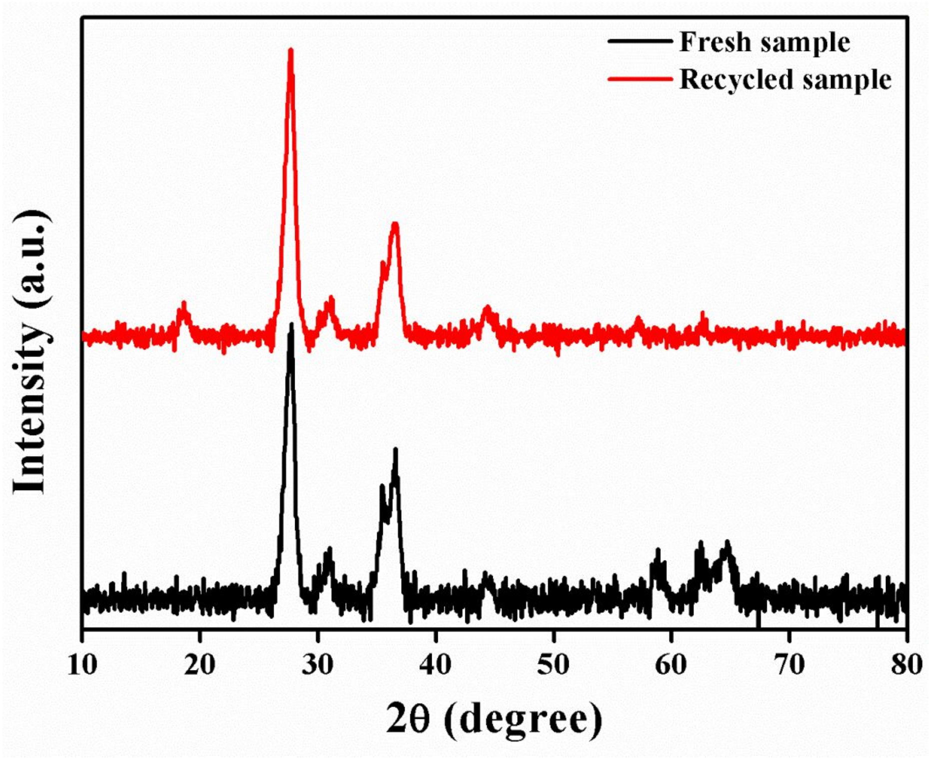

Figure S2. XRD pattern of pure and recycles $FeCo_2O_4$/g-C_3N_4 nanocomposite

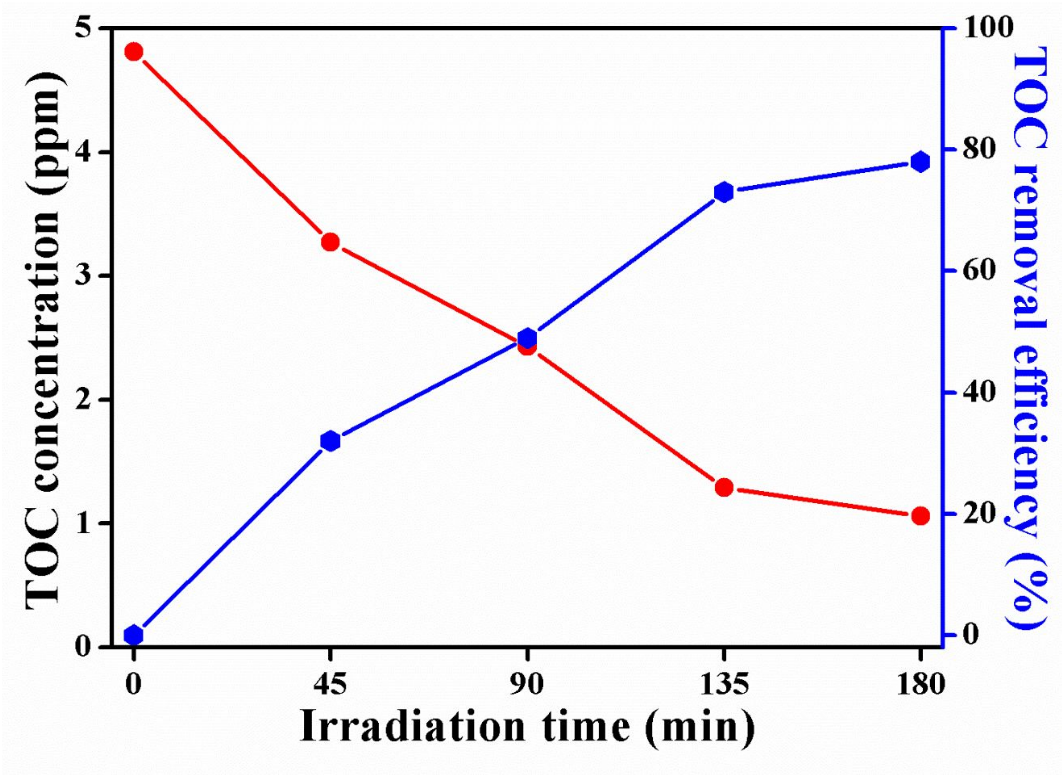

Figure S3. TOC analysis for naproxen degradation

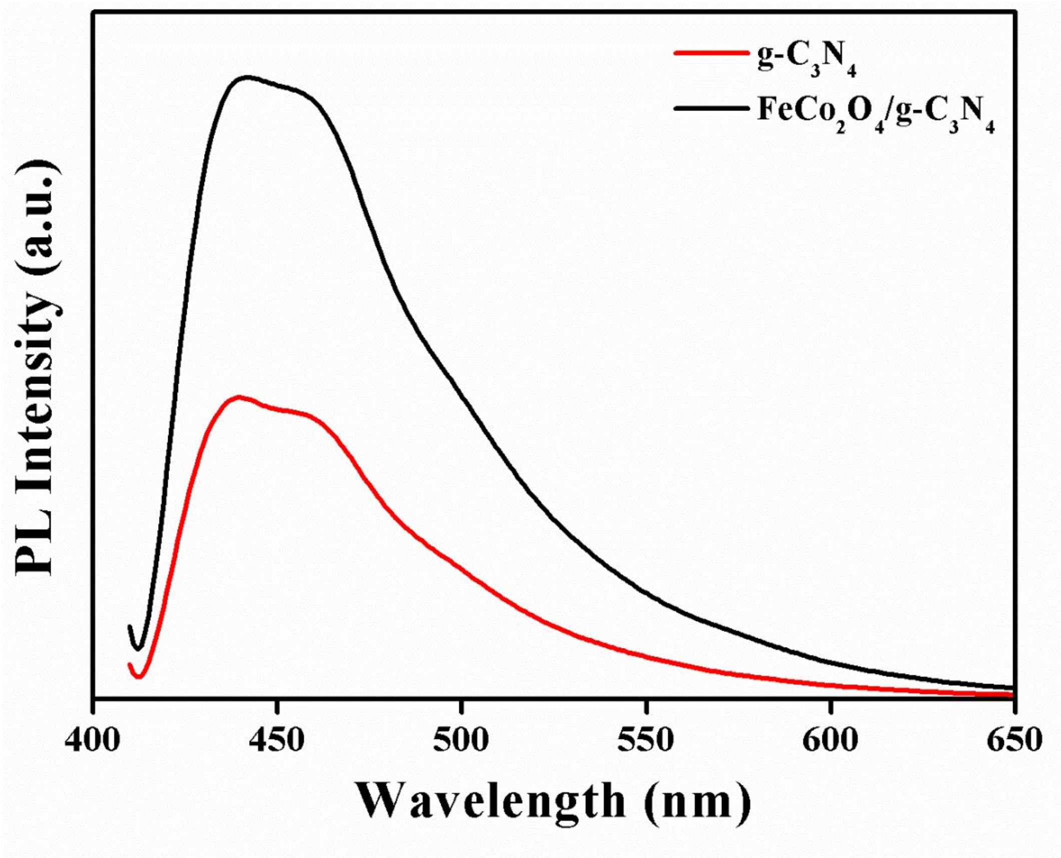

Figure S4. PL spectrum

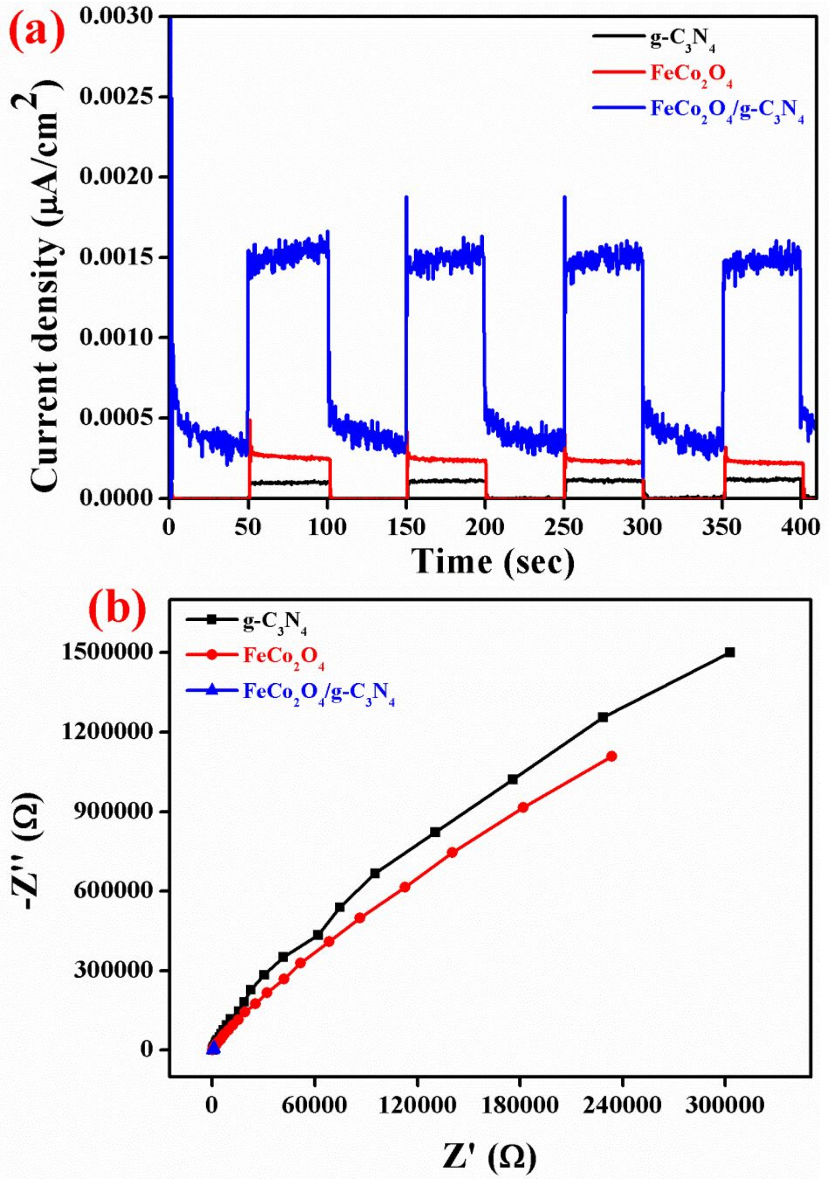

Figure S5. (a) Photocurrent and (b) Impedance analysis

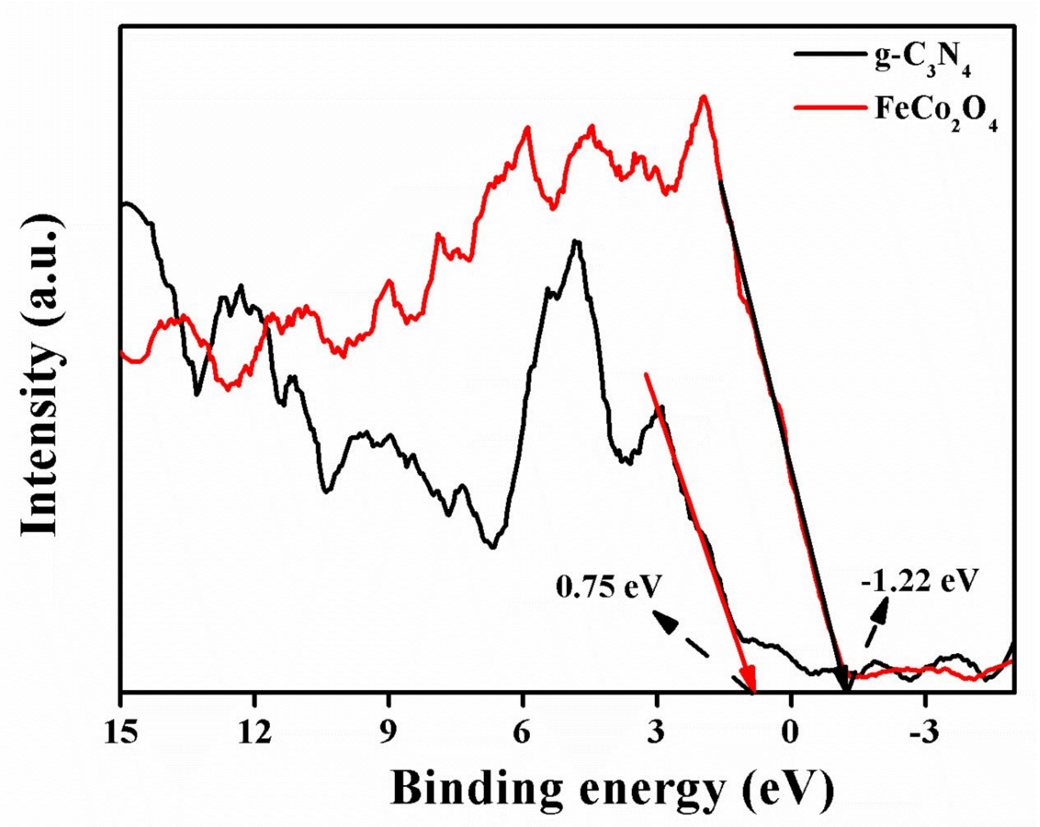

Figure S6. Valance band XPS of g-C_3N_4, and $FeCo_2O_4$
